# Topographic anatomy of the lateral surface of the parietal lobe and its relationship with white matter tracts

**DOI:** 10.3389/fnana.2024.1458989

**Published:** 2024-10-02

**Authors:** Volkan Oğlin, Ömer Orhun, Alfredo Quiñones-Hinojosa, Erik H. Middlebrooks, Orhun Mete Çevik, M. İmre Usseli, Mustafa Güdük, M. Emin Aksoy, M. Necmettin Pamir, Baran Bozkurt

**Affiliations:** ^1^Neuroanatomy Laboratory, Acıbadem University School of Medicine CASE, Istanbul, Türkiye; ^2^Department of Neurosurgery, Marmara University School of Medicine, Istanbul, Türkiye; ^3^School of Medicine, Acıbadem University, Istanbul, Türkiye; ^4^Department of Neurosurgery, Mayo Clinic, Jacksonville, FL, United States; ^5^Department of Radiology, Mayo Clinic, Jacksonville, FL, United States; ^6^Department of Neurosurgery, Acıbadem University School of Medicine, Istanbul, Türkiye

**Keywords:** parietal lobe, cortical anatomy, white matter anatomy, white matter dissection, tractography

## Abstract

Aim of this study was to define sulcal and gyral variations of the lateral parietal cortex and underlying white matter tracts and emphasize the importance of relationship between topographic anatomy of parietal lobe and white matter tracts underlying it in approaches to deep parietal and atrial lesions. Twenty-eight formalin-fixed cerebral hemispheres of 14 adult cadavers were used. Ten hemispheres were dissected from lateral to medial by fiber dissection and all stages were photographed. Our anatomic findings were supported by MRI tractography. Postcentral sulcus and intraparietal sulcus were continuous in most of the cadavers (71% in right, 64% in left side). Intermediate sulcus of Jensen was in bayonet shape in 86 and 50 percent of cadavers at right and left side, respectively. The range of perpendicular distance between the meeting point and interhemispheric fissure was 2.5–4.9 cm in right and 2.8–4.2 cm in left hemisphere whereas the range of distance between meeting point and the sylvian fissure was 3–6 cm and 2.5–5.6 in left and right hemispheres, respectively. When the meeting point was located more laterally, the probability of damaging the arcuate fasciculus and superior longitudinal fasciculus II during dissection was increased. We also found that the intraparietal sulcus and intermediate sulcus of Jensen were associated with the superior longitudinal fasciculus II, middle longitudinal fasciculus, inferior frontooccipital fasciculus, tapetum, and optic radiation. These variations and their relation to subcortical tracts should be considered in atrium and deep parietal lobe surgeries.

## Highlights

- Recognition of the variations and relations in topographic and subcortical anatomy of parietal lobe is crucial point for selecting safer surgical trajectories and minimizing the incidence of complications with a focus on preoperative planning. Postmortem fiber dissection technique and diffusion tractography imaging (DTI) reveals parietal lobe’s cortical and subcortical relation in human brain.
- In our study, the topographic anatomy of the parietal lobe and the relationship of the sulci with the subcortical structures were examined in detail using a combination of fiber dissection technique and magnetic resonance (MRI) tractography.

## Introduction

An in-depth understanding of the topographic variations in hemispheric cortical anatomy is crucial for the neurosurgeon to navigate the brain safely and effectively. Such navigation is more complex in the parietal lobe compared to other lobes due to its high intrinsic sulcal/gyral variation and due to the less-well defined anatomical landmarks. The parietal lobe occupies a very central hemispheric location neighboring all the other (frontal, temporal, occipital, insular, and limbic) lobes as well as most major long association-, comissural-and projection-tracts and the ventricular system (Yagmurlu et al., 2015). Tumors or other lesions of the region are common and there is still room for improvement of lateral surgical approaches to the parietal lobe to make them safer and more effective.

Classical anatomical studies have provided valuable information on cortical surface anatomy of the parietal lobe (Ribas, 2010). More recent ones have focused on white mater functional connectivity (Dziedzic et al., 2021). Hereby we aimed to correlate variations in cortical surface anatomy with the underlying intricate white mater connectivity. Anatomical relationships were further demonstrated using MRI tractography. This process served as a ground truth for our dissections and analysis of the topographical relationship between cortical structures and white matter tracts.

## Materials and methods

### Cadaver dissections

Institutional Review Board approval was obtained and the need for consent was waived in this retrospective cadaveric study. Twenty-eight formalin-fixed cerebral hemispheres of 14 adult cadavers were used. Rhoton’s technique was used for white matter fiber dissection. First decortication was done with the use of dissectors. U-fibers were exposed after decortication, and they were also removed with a hook. After removal of the U-fibers association fibers were dissected from lateral to medial. Klingler method was used as described (Türe et al., 2000). All specimens were fixed in 10% formalin solution for at least 2 months before dissection. Between dissection periods, specimens were kept in a 10% formalin solution. Arachnoid mater and cerebral vessels were carefully peeled away to expose the sulci on the lateral surface of each hemisphere with the aid of a Zeiss OPMI Vario 700 Surgical Microscope. Predefined gyral and sulcal distances were measured with the aid of millimetric plastic rulers. Sulcal variations were reported with previously defined terminology. Percentages and means were calculated for categorical and continuous variables, respectively. Fiber dissections were performed in 10 hemispheres from lateral to medial in a stepwise manner. Each specimen and all stages of the dissections were recorded photographically with a Canon 5D Mark II high-resolution digital camera (Canon Co., Tokyo, Japan).

### MR tractography

MR tractography was performed using a group-averaged dataset based on 1,065 subjects from the Human Connectome Project^1^ open-source dataset consisting of healthy controls ranging from 22 to 37 years of age. The preprocessed datasets available from the Human Connectome Project were used and consisted of diffusion data using a multishell diffusion scheme with b-values of 1,000, 2,000, and 3,000 s/mm^2^ and 90 directions per b-value. The spatial resolution was 1.25 × 1.25 × 1.25 mm. Diffusion data were aligned in template space and resampled to 1 mm isotropic using cubic spline interpolation followed by reconstruction (Yeh and Tseng, 2011) using generalized q-sampling imaging with a 1.7 diffusion sampling length ratio (Yeh et al., 2010). The subjects were normalized to Montreal Neurological Institute template space and averaged across all subjects. Tracts were generated in DSI Studio^2^ using a template-based automatic reconstruction described by Yeh (Yeh et al., 2010) Cortical and gyral regions-of-interest were labeled using FreeSurfer.^3^

## Results

### Sulcal variations

A summary of sulcal variations of the parietal lobe can be found in Table 1.

**Table 1:** Cortical landmarks and related anatomical characteristics.

		Left (*n* = 14)	Right (*n* = 14)
Postcentral sulcus	Course		
	Continuous	9 (64%)	10 (71%)
	Interrupted	5 (36%)	4 (29%)
	Segment Numbers		
	2 Segments	3 (21%)	4 (29%)
	3 Segments	1 (7%)	1 (7%)
	Medial course		
	Present	5 (36%)	5 (36%)
	Absent	9 (34%)	9 (64%)
	Opening to Sylvian fissure		
	Connected	9 (64%)	8 (57%)
	Not Connected	5 (36%)	6 (43%)
Marginal sulcus	Lateral surface variation		
	Present	12 (86%)	10 (71%)
	Absent	2 (14%)	4 (29%)
Parietooccipital sulcus	Medial course		
	Present	14 (100%)	14 (100%)
	Absent	0 (0%)	0 (0%)
	Lateral surface variation		
	Bayonet	8 (57%)	6 (43%)
	Arcuate	2 (14%)	4 (29%)
	T or Y	4 (29%)	2 (14%)
	Ramified	0 (0%)	2 (14%)
	Parietooccipital arch		
	Present	13 (93%)	8 (57%)
	Absent	1 (7%)	6 (43%)
Intraparietal sulcus	Course		
	Continuous	9 (64%)	10 (71%)
	Interrupted (2 segments)	5 (36%)	4 (29%)
	Opening to postcentral sulcus		
	Connected	14 (100%)	14 (100%)
	Not Connected	0 (0%)	0 (0%)
	Connection with Intermediate Sulcus of Jensen		
	Present	5 (36%)	4 (29%)
	Absent	9 (64%)	10 (71%)
Intermediate sulcus of Jensen	Course		
	Bayonet	7 (50%)	12 (86%)
	Arcuate	5 (36%)	1 (7%)
	T or Y	2 (14%)	1 (7%)
	Ramified	2 (14%)	1 (7%)
	Continuity with		
	STS	9 (63%)	8 (56%)
	IPS	1 (7%)	1 (7%)
	Distance to meeting point	Interhemispheric Fissure	2.8–4.2	2.5–4.9
Sylvian Fissure		2.5–5.6	3–6

IPS, intraparietal sulcus; STS, superior temporal sulcus.

#### Postcentral sulcus

**Figure 1:**
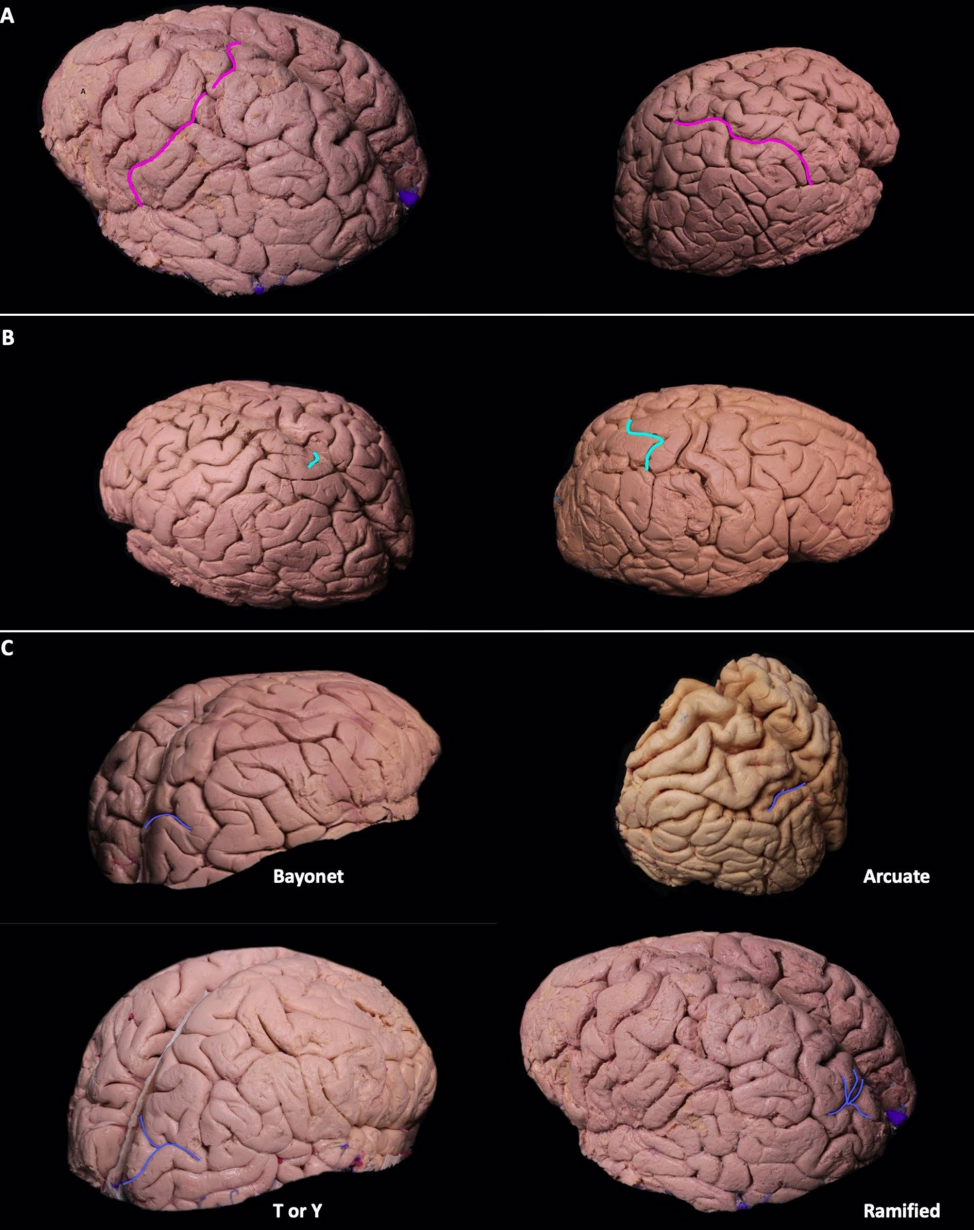

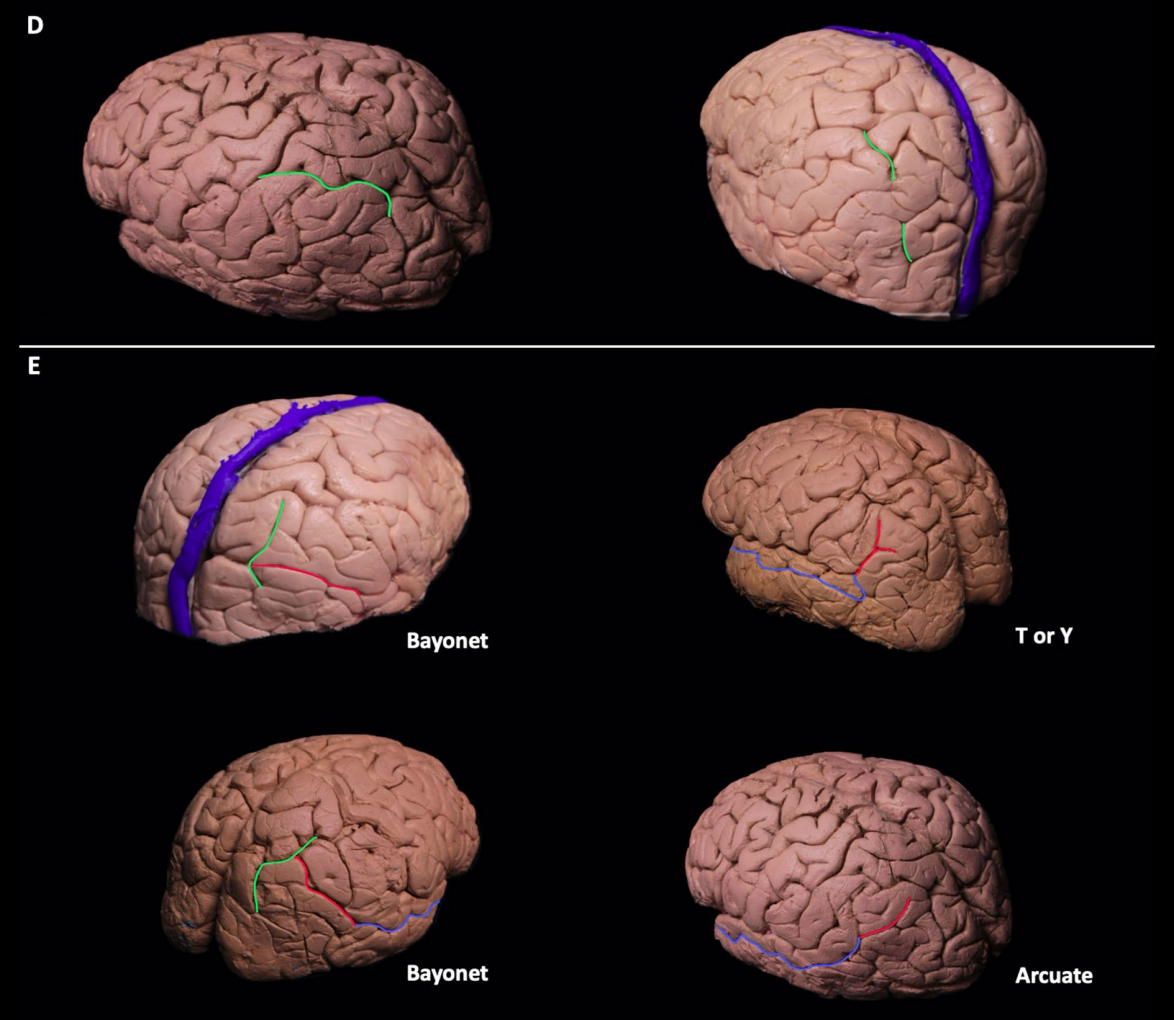
**(A-C)** Sulcal variations of the dorsolateral parietal cortex. Postcentral sulci in two different hemispheres can be seen in pink. The postcentral sulcus can be double or single segmented **(A)**. Superolateral extension of the marginal sulcus can be seen in turquoise. Its extension can be short/absent, or it can extend to the superolateral parietal surface **(B)**. Patterns of parietooccipital sulcus can be seen in blue. Parietooccipital sulcus can be in arcuate form, bayonet form, Y form, or ramified form **(C)**. **(D-E)** The course of intraparietal sulcus can be seen in green. It can be continuous without segmentation, or it can have two segments **(D)**. The intermediate sulcus of Jensen (red) can be the continuation of the intraparietal sulcus seen in green, superior temporal sulcus seen in blue, or the continuation of both the superior temporal and intraparietal sulcus. The intermediate sulcus of Jensen can be in arcuate form [bayonet form, or in T or Y form **(E)**].

The postcentral sulcus (POCS) is a sulcus of the parietal lobe that separates the postcentral gyrus (the primary somatosensory cortex) from the remainder of the parietal lobe and the secondary somatosensory cortex. The POCS was identified as a single continuous segment in 64% (9/14) of the left and 71% (10/14) of the right hemispheres, and as two separate, discontinuous segments in 21% (3/14) of the left and 29% (4/14) of the right hemispheres. Three segmented POCS were observed only once on the left and right hemispheres 7% (1/14). In 36% (5/14) of all cadavers, it extended to the medial surface on both hemispheres. In 64% (9/14) of left and 57% (8/14) of right hemispheres, the sylvian fissure opened into the POCS (Figure 1A).

#### Superolateral extension of the marginal sulcus

The marginal sulcus, also known as “pars marginalis” or “ramus marginalis,” is the extension of the cingulate sulcus and separates the paracentral lobule from the precuneus of the parietal lobe on the medial surface. In 86% (12/14) of left and 71% (10/14) of right hemispheres, the marginal sulcus extended to the superolateral surface. In 63% (9/14) of cadavers, the superolateral extension was present bilaterally (Figure 1B).

#### Superolateral surface of parieto-occipital sulcus

The parieto-occipital sulcus (POS) marks the boundary between the cuneus and precuneus and lies between the parietal and occipital lobes. The POS courses inferiorly from the vertex on the medial surface of the hemisphere. A short segment of POS extended onto the lateral surface of the parietal hemisphere in all cadavers. Various forms of the POS, in which it extends to the superolateral surface, were observed. Bayonet form was found in 57% (8/14) and 43% (6/14), arcuate form in 14% (2/14) and 29% (4/14), T or Y in 29% (4/14) and 14% (2/14), and ramified form in 0% (0/14) and 14% (2/14) of left and right hemispheres, respectively. Parietooccipital arch is defined as a gyral bridge between the occipital and parietal lobe observed on the dorsolateral surface of the hemisphere. It is seen when the lateral extension of POS is not in contact with the intraparietal sulcus (Ribas, 2010). In the right hemisphere, 56% (8/14) had the parietooccipital arch, while 43% (6/14) showed no arch, and the POS was associated with the intraparietal sulcus. In the left hemisphere, 93% (13/14) had the parietooccipital arch, while 7% (1/14) had no arch and the POS was associated with the intraparietal sulcus (Figure 1C).

#### Intraparietal sulcus

The intraparietal sulcus (IPS) is located on the lateral surface of the parietal lobe. It divides the posterior parietal cortex into superior and inferior parietal lobules (SPL and IPL, respectively). The IPS intersects with and ends at the POS. Posteriorly, it courses at the junction of the SPL and IPL. Although its orientation is described quite variably, it generally runs somewhat parallel to the interhemispheric fissure.

The IPS was identified as a single continuous segment in 64% (9/14) of the left and 71% (10/14) of the right hemispheres. In 36% (5/14) of the left and 29% (4/14) of the right hemispheres, it was observed as two separate segments and discontinuous segments, respectively. In all cadavers, the IPS connected to the POS (Figure 1D).

#### Intermediate sulcus of Jensen

The intermediate sulcus of Jensen (ISJ) divides the IPL into the supramarginal (SmG) and angular gyri (AnG). It is either the continuation of the IPS and/or the superior temporal sulcus (STS). The form of the ISJ was determined to be shaped as a bayonet in 50% (7/14) and 86% (12/14), arcuate in 36% (2/14) and 7% (1/14), and T or Y in 14% (2/14) and 7% (1/14) of the left and right hemispheres, respectively. The ISJ was the continuation of the STS in 63% (9/14) of the STS and IPS in 28% (4/14), and of the IPS in 7% (1/14) of the left hemispheres. In the right hemispheres, results were 56% (8/14), 36% (5/14), and 7% (1/14) of the STS, the STS and IPS, and the IPS, respectively (Figure 1E). Three different sulcal configurations of ISJ have deterministic effect on the anatomy of angular and supramarginal gyrus. If ISJ is connected to both STS and IPS there is no gyral bridge between angular and supramarginal gyrus, if ISJ is connected to STS but not IPS there is a superiorly located gyral bridge between angular and supramarginal gyrus whereas if ISJ is connected to IPS but not STS there is an inferiorly located gyral bridge between angular and supramarginal gyrus.

#### Meeting point of the postcentral sulcus and intraparietal sulcus

**Figure 2:**
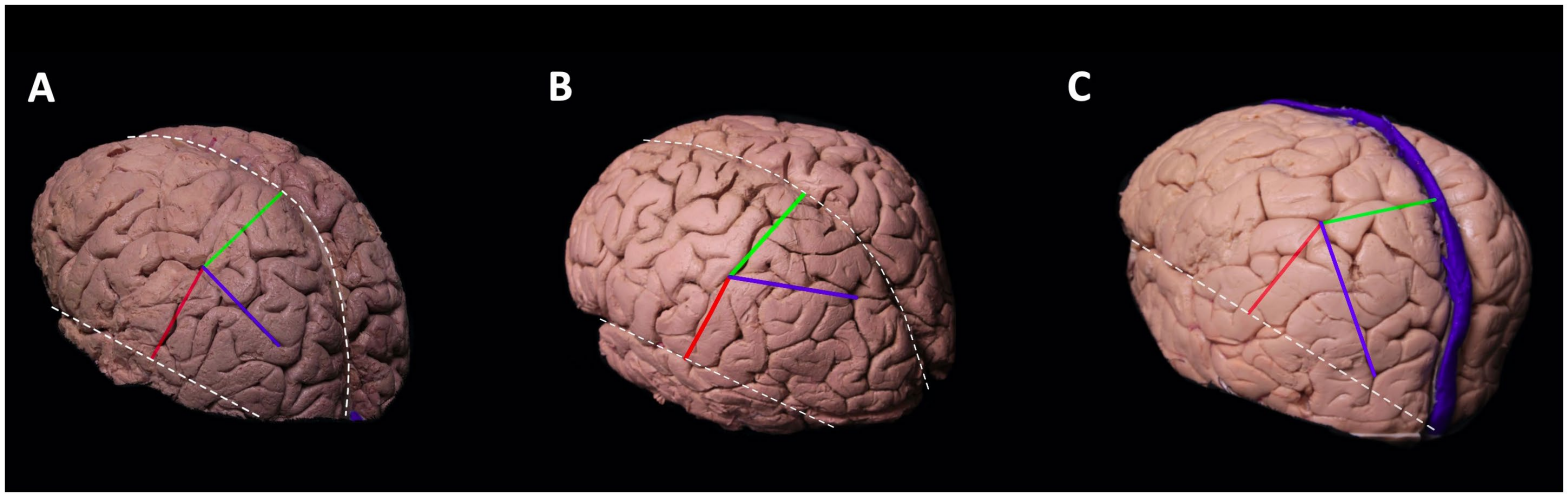
Meeting point of the postcentral sulcus and intraparietal sulcus. Cortical view of the left hemisphere.The blue line depicts the course of intraparietal sulcus, red line is the perpendicular distance between the meeting point and sylvian fissure, and the green line depicts the perpendicular distance between meeting point and interhemispheric fissure. Superiorly located white dotted line is the interhemispheric fissure and the inferiorly located white dotted line is the sylvian fissure. The course of the intraparietal sulcus determines the localization of the meeting point. The intraparietal sulcus can course parallel to interhemispheric fissure **(A)**, deviate laterally **(B)**, or deviate medially **(C)**. If the intraparietal sulcus deviates laterally, then the perpendicular distance between the meeting point and interhemispheric fissure increases **(B)**. If it deviates medially, then the distance decreases **(C)**.

The IPS and PCS intersected in all cadavers. This point is known as the intraparietal point (Ribas et al., 2006). The distance between this meeting point perpendicular to the interhemispheric fissure and the sylvian fissure was calculated in the left hemisphere to be 2.8–4.2 cm, and in the right hemisphere 2.5–4.9 cm. The distance from the meeting point to the sylvian fissure in the left hemisphere was between 2.8–5.6 cm and 3–6 cm in the right (Figure 2; Table 1).

### Subcortical connections of the temporoparietal junction and their relationship to gyral and sulcal anatomy

**Figure 3:**
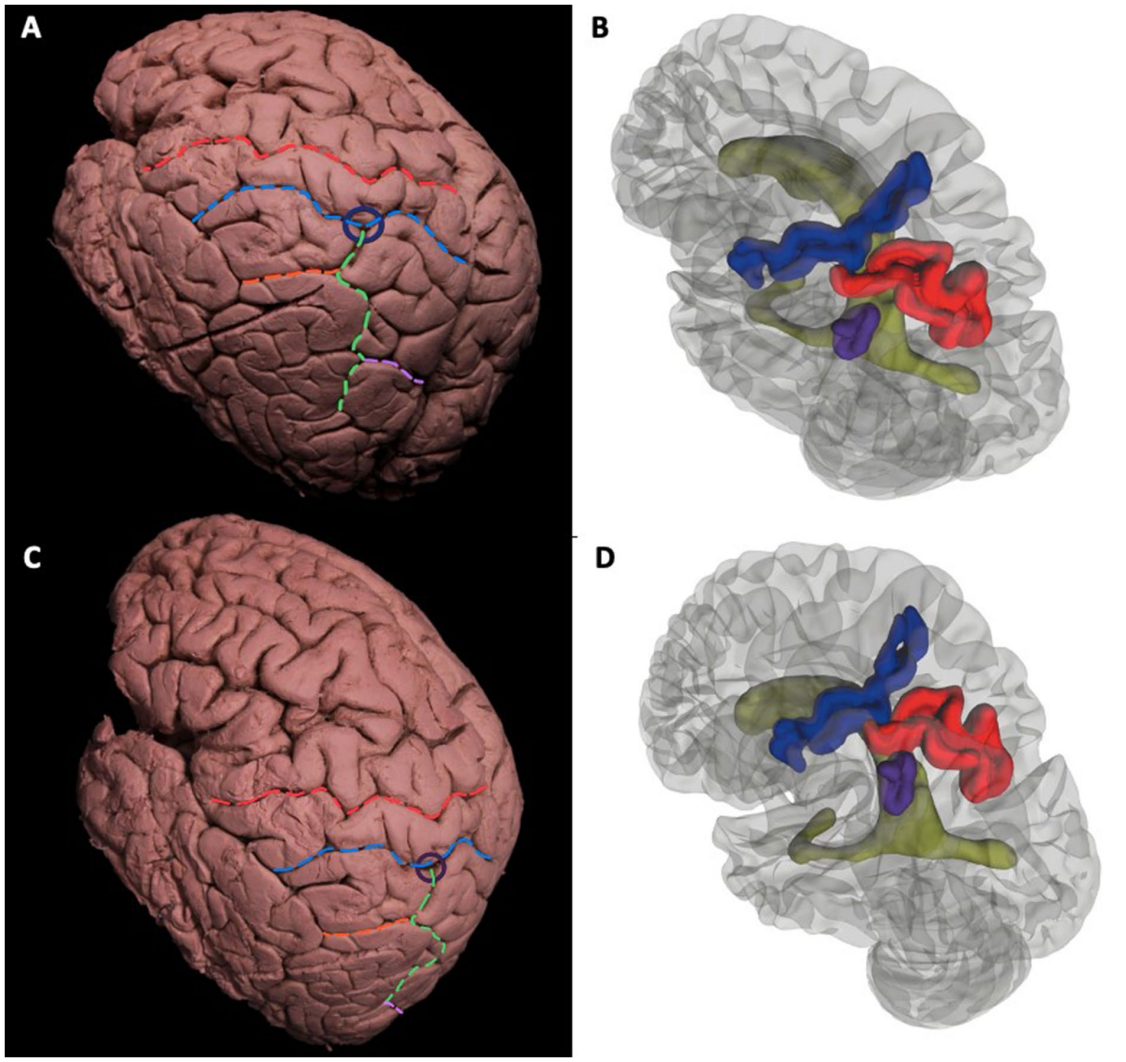
General description of gyri and sulci of parietal lobe. Cortical view of the left hemisphere. Surface landmarks of the dorsolateral left cerebral hemisphere **(A-D)**. Superodorsolateral view of cadaveric dissection **(A)** and 3D rendered MRI **(B)** Red dotted line indicates the central sulcus that separates the precentral and postcentral gyrus. Blue dotted line is the Postcentral Sulcus. Green dotted line is the Intraparietal Sulcus that further divides the Posterior Parietal Lobule into Süperior Parietal Lobule and Inferior Parietal Lobule. Orange dotted Intermediate of Jensen Sulcus delineates the two parts of the Inferior Parietal Lobule as Supramarginal Gyrus and Angular Gyrus. Purple line is the Parietoocipital Sulcus. Intermediate of Jensen Sulcus is the continuation of the Intraparietal Sulcus. Parietooccipital Sulcus intersected with Intraparietal Sulcus.The Intraparietal Sulcus and Postcentral Sulcus intersected in all cadavers that is called Meeting Point and shown with blue circle **(A)**. Relationship of sulci with ventricle; Blue, red, purple, and green gyral parts corresponds to Postcentral Sulcus, Intraparietal Sulcus, Intermediate of Jensen Sulcus and lateral ventricle, respectively. Intersections of Sulci (meeting point) can be crucial for surgical approach **(B)**. Lateral view of cadaveric dissection **(C)** and 3D rendered MRI **(D)**. Red Dotted Line = Central Sulcus, Blue Dotted Line = Postcentral Sulcus, Green Dotted Line = Intraparietal Sulcus, Orange Dotted Line = Intermediate Sulcus of Jensen, Pink Dotted Line = Parietooccipital Sulcus, Parliament Circle = Meeting point (of Intermediate Sulcus of Jensen and Postcentral Sulcus). Blue Gyrus = Postcentral Sulcus, Red Gyrus = Intraparietal Sulcus, Purple Gyrus = Intermediate Sulcus of Jensen, Green: Left Lateral Ventricle.

General description of gyri and sulci of parietal lobe can be found in Figures 3A–D. The postcentral sulcus divides the postcentral gyrus and posterior parietal lobule. The intraparietal sulcus that further divides the posterior parietal lobule into superior parietal lobule and inferior parietal lobule. The intermediate part of Jensen Sulcus divides the two parts of the inferior parietal lobule as the supramarginal gyrus and the angular gyrus. The intraparietal sulcus and postcentral sulcus intersected in all cadavers that is called meeting point (Figures 3A–D). The meeting point is the closest point to the atrium and is an important landmark in the surgical approach to the atrium. Intermediate of Jensen sulcus is the continuation of the intraparietal sulcus. Parietooccipital sulcus intersected with intraparietal sulcus.

Stepwise dissections from lateral to medial were done to understand the relationship between sulcal variations and white matter tracts. After decortication of grey matter and dissecting U-fibers, association fibers at the temporo-parietal junction were revealed.

**Figure 4:**
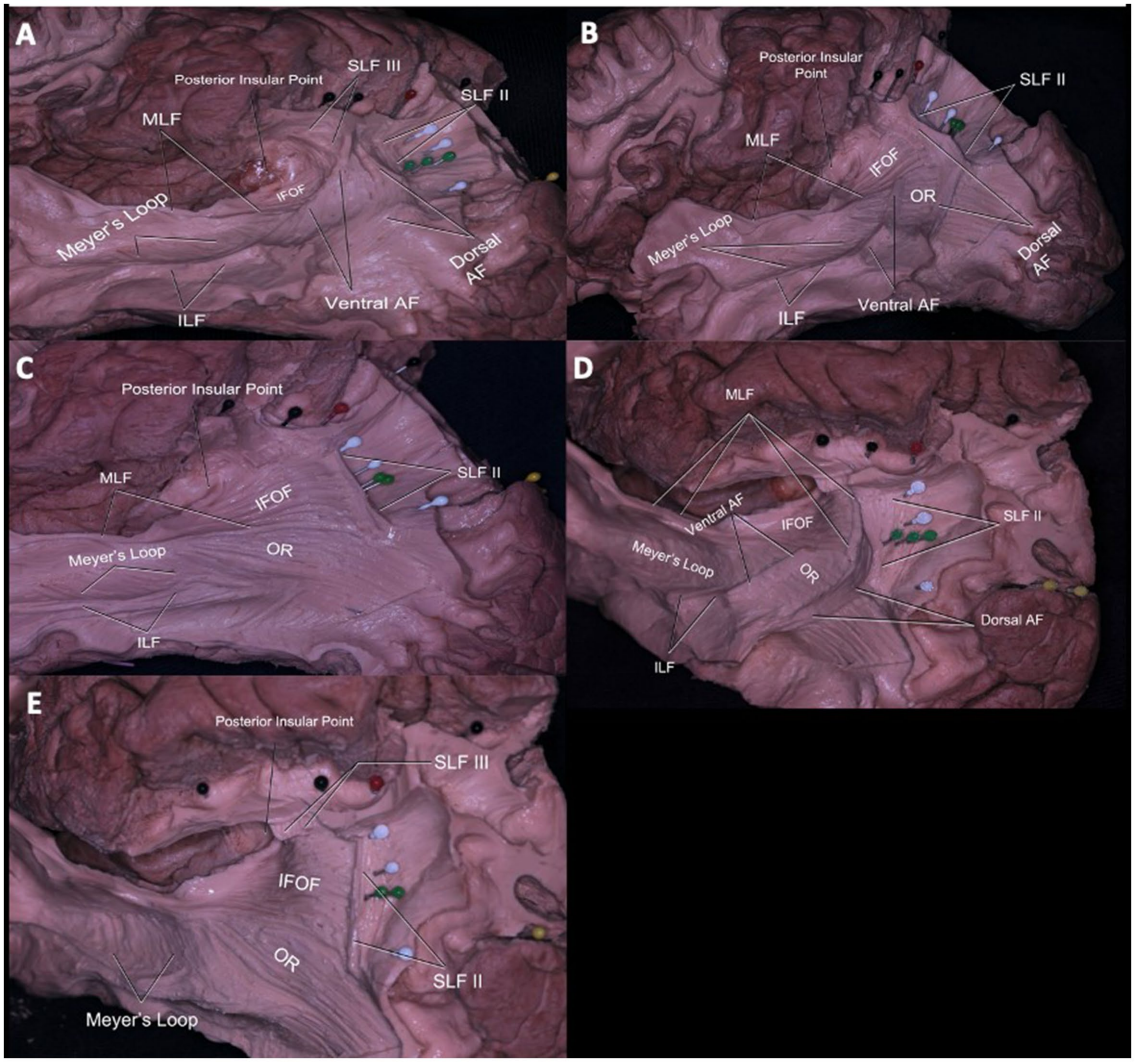
Subcortical Connections of the Temporoparietal Junction and Their Relationship to Gyral and Sulcal Anatomy. Relationship of the white matter tracts of the lateral parietal lobe **(A–E)**. The SLF III, AF, and SLF II coursed deep to the postcentral sulcus (black pins) from lateral to medial. AF fibers course inferolateral to the ISJ and curve anteriorly at the midpoint between ISJ (green pins) and posterior insular point. Arising from superior and middle temporal gyrus, fibers of MdLF course deep to the fibers of AF at the point where the fibers of AF curve and reach to superior parietal lobule. Deep to the fibers of MdLF, branches of IFOF are seen connecting frontal and occipital gyri **(A)**. AF fibers course inferolateral to ISJ (green pins) and IPS (white pins). Fibers of OR move inferomedial to the fibers of IFOF and MdLF, thus OR fibers do not course deep to the IPS (white pins). Deep to MdLF (dissected) fibers of IFOF can be seen **(B)**. AF is totally dissected to demonstrate parts of the sagittal striatum including MdLF, ILF, IFOF, OR from lateral to medial. Only the inferior most portion of the MdLF is left to demonstrate IFOF. Normally, MdLF course over the IFOF. Fibers of MdLF course parallel and deep to the ISJ (green pins) and terminate in the angular gyrus. Fibers of IFOF move deep and parallel to MdLF coursing to the inferior and superior occipital lobe **(C)**. Superolateral view of the left hemisphere PCS (black pins), IPS (white pins), ISJ (green pins), meeting point of PCS and ISJ (red pin) and their relationship to underlying white matter can be seen. Fibers of SLF II can be seen coursing deep and parallel to IPS. Fibers of SLF II and AF course parallel together, while fibers of MdLF and IFOF can be found in the deeper layer. The SLF II passed beneath the IPS just deep to the sulcus along the anterior bank of the ISJ to reach in the AnG **(D)**. The SLF II fibers run parallel to the IPS and perpendicular to the ISJ. Deeper, the IFOF fibers run parallel to the ISJ and perpendicular to the IPS. The OR is not related to the meeting point, and only a minor part of it was related to the caudal end of the IPS **(E)**. Red pin = Meeting point (of PCS and IPS), White Pin = IPS, Green Pin = ISJ, Black Pin = PCS, Yellow Pin = POS. SLF = Superior Longitudinal Fasciculus, MdLF = Middle Longitudinal Fasciculus, IFOF: Inferior frontooccipital fasciculus, AF: Arcuate fasciculus, OR: Optic radiation, ILF: Inferior longitudinal fasciculus.

The most superficial long association fibers were the arcuate fasciculus (AF) portions of the superior longitudinal fasciculus (SLF), which has three parts. We focused our attention on SLFII and III since SLFI is not accessible from a lateral dissection.The SLFII extended from the occipital peristriate cortex to the dorsolateral prefrontal cortex and lateral frontopolar cortex. The SLF III and AF were exposed in the frontoparietal operculum (Figure 4A). The SLF III was located ventrally and laterally to the SLF II and runs from the SmG to the pars opercularis of the frontal lobe.

The AF is divided into ventral(vAF) and dorsal (dAF) segments. Fibers of the vAF arise from the mid-posterior part of the superior temporal gyrus (STG) and middle temporal gyrus (MTG) and, via the SmG, reached the pars opercularis with the SLF III horizontally. Nevertheless, the dAF started from posterior to the MTG and inferior temporal gyrus (ITG) via the AnG, and reached to pars opercularis with the SLF II (Figure 4A).

When the AF was dissected completely, components of the sagittal stratum were seen, including the inferior longitudinal fasciculus (ILF), middle longitudinal fasciculus (), inferior fronto-occipital fasciculus (IFOF), and optic radiation (OR). Parts of the sagittal stratum are organized as a continuum of white matter tracts and form a sheet-like structure. The superficial part of the sagittal stratum is composed of MLF and ILF, the middle part by IFOF, and the deep layer by OR (Figures 4B,C). At the infrasylvian area, the main long association fiber pathways are the MLF and ILF. Fibers of the MLF originated from the superior temporal gyrus and middle temporal gyrus and terminated at the AnG. The ILF fibers extended from the dorsolateral occipital cortex, through the inferior temporal gyrus, and termination the temporal pole (Figures 4A–C).

Fibers of the IFOF were visible after removal of the MLF. The inferior most fibers of the MLF were left intact to demonstrate the relationship between the MLF and IFOF (Figure 4C). The IFOF fibers extended anteriorly to the prefrontal cortex and posteriorly to occipital lobe along the lateral wall of the temporal and occipital horns.

Meyer’s loop originates from the lateral geniculate body and extended to the anterior temporal lobe and finally reached the occipital visual cortex as part of the OR (Figure 4C). The OR crosses AF fibers at a right angle and lies deep and medial to the AF (Figure 4B).

### Sulci and white matter relationship

#### Postcentral sulcus and its relationship with white matter tracts

In the sagittal plane, the SLF III, AF, and SLF II coursed deep to the postcentral sulcus from lateral to medial. The SLF II and SLF III, along with the AF, proceeded beneath the PCS (Figures 4A,D). The SLF III progressed at the level of the inferior frontal gyrus and the SLF II at the level of the middle frontal gyrus. The vAF extended medial to the SLF III, and the dAF extended ventral to the SLF II deep to the postcentral sulcus.

#### Intraparietal sulcus and its relationship with white matter tracts

From superolateral to inferomedial, the SLF II and MLF were the most superficial fibers coursing beneath the IPS (Figures 4A,C,D). Fibers of the AF were also located superficially at the same level with the SLF II, but they were located lateral to the IPS (Figure 4D). The SLF II passed beneath the IPS just deep to the sulcus before turning superiorly along the anterior bank of the ISJ to reach in the AnG (Figure 4D). Fibers of the MLF originated from the superior temporal gyrus and middle temporal gyrus and terminated at the AnG, coursed posteromedial to the ISJ and along the lateral aspect of the posterior portion of the IPS (Figures 4B,C). Deep to these fibers, IFOF fibers were found. Fibers of the OR coursed parallel to the IFOF fibers and were located lateral to the IPS (Figure 4C). The IFOF branches to the SPL were along the medial bank of the IPS. The OR is not related to the meeting point, and only a minor part of it was related to the caudal end of the IPS (Figures 4C,D).

#### Intermediate sulcus of Jensen and its relationship with white matter tracts

The SLF II, MLF, and IFOF were found underlying the ISJ as the IPS. The main difference between the ISJ and IPS was the presence of AF and SLF III fibers inferolateral and anterolateral to the ISJ, respectively (Figures 4B,D).

Fibers of the AF passed inferolateral to the ISJ and curved anteriorly at the midpoint between the ISJ and posterior insular point (Figures 4A,D). MLF fibers that connect the AnG passed posterior medial to the ISJ and along the lateral aspect of the posterior portion of the IPS (Figures 4C,D). Fibers of the OR coursed inferomedial to fibers of the IFOF and MLF; thus, OR fibers did not pass deep into the ISJ (Figures 4C,D,F).

#### Atrium of the lateral ventricle and its relationship with white matter tracts

**Figure 5:**
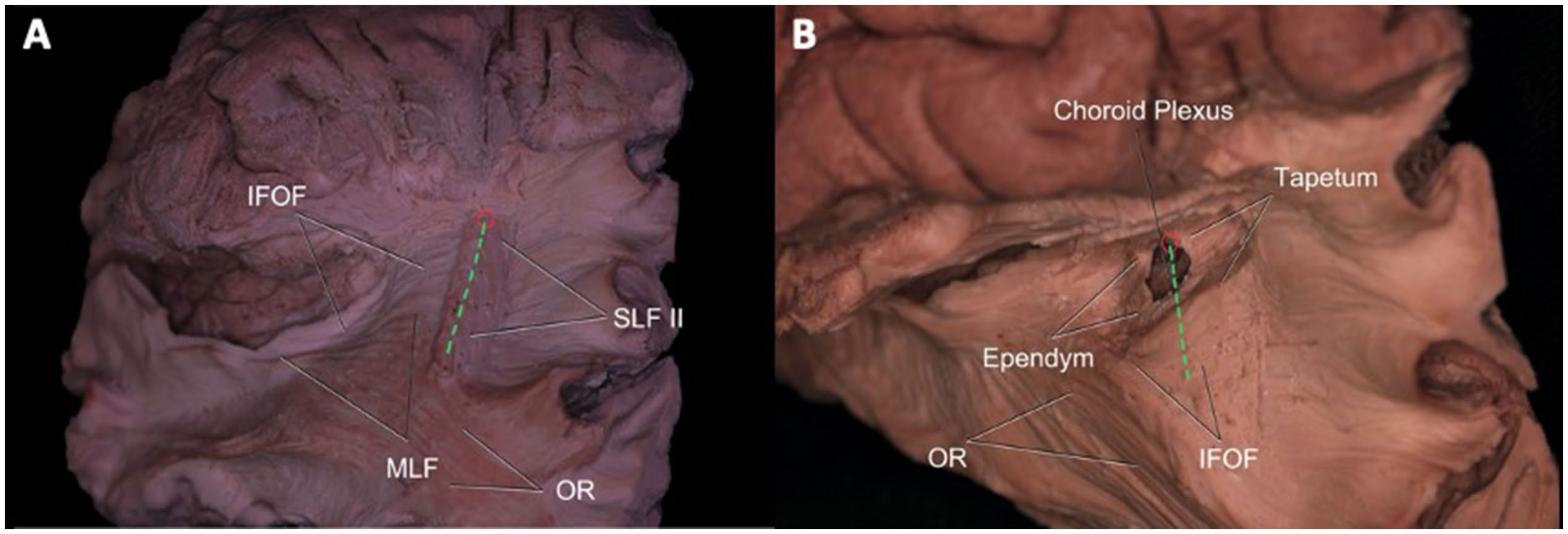
Atrium of the Lateral Ventricle and Its Relationship with White Matter Tracts. Superolateral view of the left hemisphere demonstrating the relationship of SLF II, IFOF, MdLF, and OR’s relationship to the intraparietal sulcus and meeting point. SLF II fibers travel beneath the IPS (green dotted line), while IFOF fibers pass medial to the fibers of SLF II crossing it in right angle. MdLF fibers normally lie superior to fibers of IFOF and course deep to the IPS. OR is not related to meeting point and only a minor part of it is related to caudal end of the IPS. An entry to the atrium from IPS may damage SLF II, IFOF, MdLF, and OR (at the distal end), whereas a cut from the meeting point would spare MdLF, OR, and AF **(A)**. After dissection of SLF II, tapetum, and ependyma, entry into the atrium of the lateral ventricle is achieved. Choroid plexus can be observed at the wall of the atrium **(B)**. Green Dotted Line = Intraparietal Sulcus, SLF = Superior Longitudinal Fasciculus, MdLF = Middle Longitudinal Fasciculus, IFOF: Inferior frontooccipital fasciculus, AF: Arcuate fasciculus, OR: Optic radiation, ILF: Inferior longitudinal fasciculus.

The meeting point is described as the closest point to the atrium. Accordingly, dissection from the meeting point and the IPS was done to demonstrate important white matter tracts in relation to the atrium. If the approach to the atrium was from the meeting point or anterior half of the IPS, SLF II, tapetum, and ependyma were seen from the most superficial to the deepest layers, respectively. When the approach was from the posterior half, the most superficial tract was again the SLF II, while a second deeper layer of white matter tracts consisted of MLF and IFOF from superior to inferior, respectively. Medial to the MLF and IFOF, the tapetum and ependyma were located at the deepest layer, forming the wall of the atrium (Figures 5A,B).

## Discussion

Among all brain lobes, the parietal lobe has the most extensive variability in sulcal and gyral anatomy (Gürer et al., 2013; Ovalioglu et al., 2018; Gonul et al., 2014). Consequently, topographic landmarks are difficult to recognize, which in turn complicates planning of surgical approaches to parietal cortical, subcortical and ventricular lesions. Approaches through this region to reach the atrium are particularly complex. Neuroanatomical studies of the sulcal/gyral patterns and their variations improve the safety of neurosurgical procedures in this area.

### Cortical variations

In our study, no segmental or extensional anatomical differences were found in POCS between the right and left hemispheres. POCS did not intersect with the sylvian fissure in 44% of the hemispheres. To our knowledge, no previous study reported POCS continuance and relation with sylvian fissure. Therefore, the possibility of mistaking the POCS with the central sulcus during surgery should be considered. The intersection of the POCS with the intraparietal sulcus may be another important landmark in distinguishing the postcentral sulcus from the central sulcus.

Besides pre- and post-central gyri, the marginal sulcus is another landmark for localizing the central lobe especially in interhemispheric approaches. In all cadavers, the central lobe located anterior to the marginal sulcus that separates precuneus from postcentral gyrus was central lobe. In almost 80% of all hemispheres, marginal sulcus was continuous on the dorsolateral surface. If this is the case, then it can be used as a landmark to determine the postcentral sulcus also in the lateral approaches. Since the POS reached the lateral surface in all hemispheres, it can be followed from the lateral to medial surface to distinguish cuneus from precuneus in the posterior interhemispheric approach. Retraction and manipulation anteriorly to the sulcus are recommended from the beginning of the inter-hemispheric dissection. Maintaining the dissection anterior to the POS should minimise the chances of injury to the calcarine sulcus and avoid a visual field defect. Therefore, it is crucial to recognise the lateral extension of the POS, remember its location posterior to the marginal sulcus, and be mindful that anatomical variations are not uncommon (Koutsarnakis et al., 2021). Therefore, it is important to recognize the lateral extension of the POS, and it should be kept in mind that it is located posterior to the marginal sulcus and has morphological variations.

Ribas et al. reports the ISJ as a sulcus opening to the STS or IPS in their cadaveric studies (Ribas, 2010). Using their description, we evaluated frequencies of these morphological patterns in 28 hemispheres. The most common shape of ISJ was bayonet followed by arcuate, T or Y, and ramified. Continuity of the ISJ with both the STS and IPS was uncommon, seen in only 29% of right hemispheres. Our results suggested that this anatomical rule cannot be definitively valid.

In radiological studies, the frequency of a meeting point of the IPS and the POCS on the lateral surface has been reported to be low (Makris et al., 2005). This can be explained by the segmental variations of the IPS and POCS: in our series the IPS was interrupted in 36% of left, and 29% of right hemispheres, whereas the POCS was interrupted in 36% of left and 29% of right hemispheres. This relatively common variation of interrupted IPS and POCS can explain the low frequency of radiologic appearance of the meeting point.

### Relation to subcortical white mater tracts

The parieto-temporal junction subcortical area is the intersection of important long association fibers. There are several studies demonstrating white matter tracts passing under gyri and sulcus of the parietal lobe, the number of studies correlating the variation in cortical anatomy with subcortical connectivity are limited (Lin et al., 2021; Burks et al., 2017). A wide range complications can be seen in this region surgery, from mild neurocognitive damage to major speech and visual field deficits. For transsulcal approaches, the relationship of sulci with subcortical white matter pathways should be well known.

**Figure 6:**
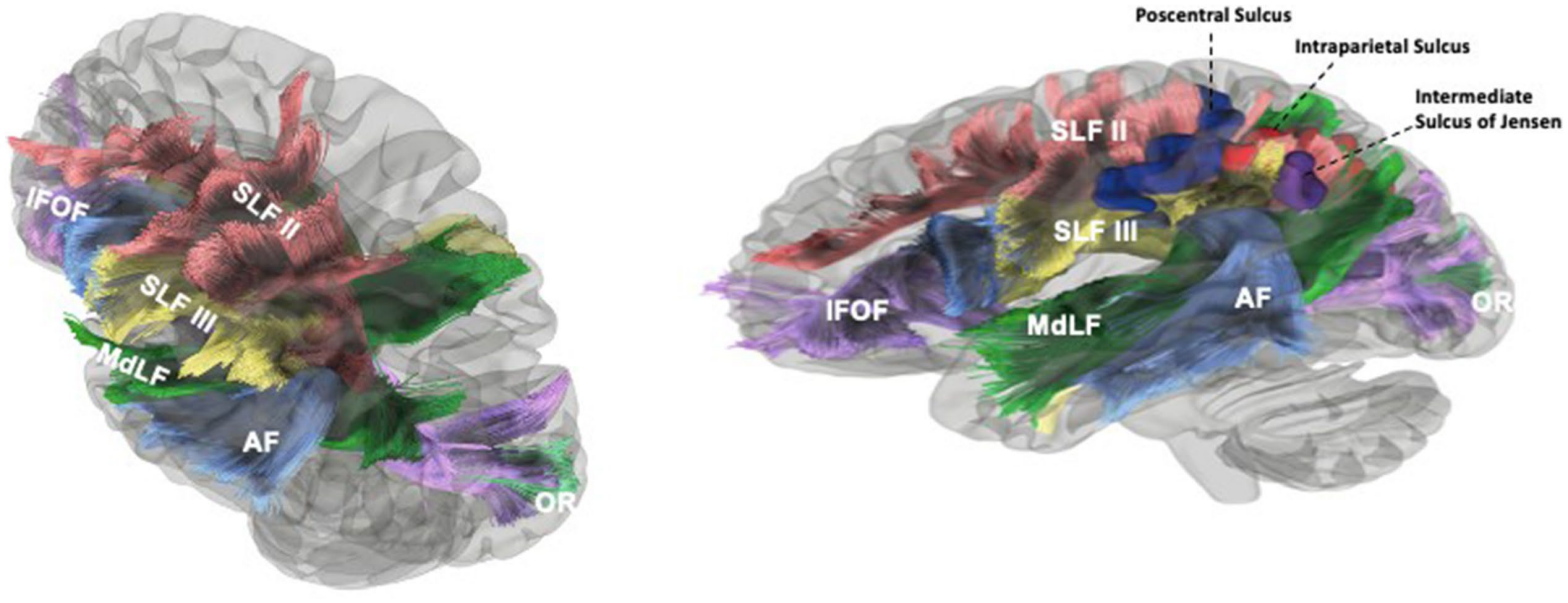
Tractographic findings of the white matter underlying the sulci of the parietal lobe. The SLFII extended from the occipital peristriate cortex to the dorsolateral prefrontal cortex and lateral frontopolar cortex. The SLF III and AF were exposed in the frontoparietal operculum. The SLF III was located ventrally and laterally to the SLF II and runs from the SmG to the pars opercularis of the frontal lobe **(A)**. SLF II posterior fibers course deep to the IPS just deep to it before turning superiorly along the anterior bank of the sulcus of Jensen to insert in then angular gyrus. AF fibers (ventral and dorsal segments) originate from the temporal gyri, turn anteriorly after reaching the supramarginal gyrus and angular gyrus, then travel medially to SLF III and ventral to SLF II to reach the frontal lobe. Medial to AF, MdLF and IFOF can be seen in temporal region. AF fibers course inferolateral to ISJ and IPS. MdLF fibers that connect the angular gyrus course posterior and medial to the ISJ and along the lateral aspect of the posterior portion of IPS. The IFOF branches to the SPL lie along medial and posterior bank of IPS. MdLF and IFOF (parts of SS) and their relationship to the ISJ can be seen **(B)**. Blue Gyrus = Postcentral Sulcus, Red Gyrus = Intraparietal Sulcus, Purple Gyrus = Intermediate Sulcus of Jensen, Yellow: Left Lateral Ventricle. Blue Fibers = AF, Green Fibers = MdLF, Violet Fibers = IFOF, Yellow Fibers = SLF III, Red Fibers = SLF II, Turquoise fibers = OR.

In the approach to the subcortical area along the post-central sulcus, there is a high probability of injury to the SLFIII, AF and SLF II fibers lateral to medial, respectively. Significant neurocognitive defects may occur due to damage to these fibers (Ribas et al., 2006; Makris et al., 2005; Pilipović-Dragović et al., 2018; Schmahmann et al., 2007). Severe speech disorders can be seen due to AF and SLF III damage, especially in the dominant hemisphere (articulatory disorders, repetition disorder and phonemic paraphasia and transcortical motor aphasia) (Yagmurlu et al., 2016; Fridriksson et al., 2010; Glasser and Rilling, 2008). In the intraparietal sulcus approach, SLF II, MLF, IFOF, OR and tapetum fibers may be damaged. Semantic paraphasia (dominant hemisphere) (Glasser and Rilling, 2008; Duffau et al., 2003; Duffau et al., 2005; Duffau et al., 2008) and visual field defect (Duffau et al., 2008) may occur due to IFOF and OR injury, respectively. In dissections where the most posterior part of the sulcus is preserved, the possibility of injury to the OR is reduced. The AF fibers turn superiorly just lateral to the IPS and the fibers are thus relatively spared. SLF II fibers are relatively less damaged if the dissection is performed parallel to the IPS. In the ISJ approach, SLF II, MLF, IFOF and tapetum fibers are most likely to be damaged. SLF III, dAF and OR fibers are not directly damaged. However, minor injury may occur due to their proximity to the ISJ. If dissection is performed parallel to the ISJ, fibers other than SLF II are relatively less damaged because dissection is performed parallel to the course of the fibers, and major complications such as semantic paraphasia may be reversible. In Table 2, important sulci of the parietal lobe including the POCS, IPS,and ISJ, and white matter tracts passing under these sulci, are described. Further information about clinical manifestations of injuries to these tracts are provided in Table 3. Besides gross anatomic evidence provided by cadaveric dissections, our study also includes tractographic evidence regarding white matter underlying sulci of the parietal lobe (Figures 6A,B). These data are based on our anatomy and DTI study results, and we think they should be considered in preoperative planning. In addition to this information, we believe that the relationship of the lesion with subcortical white matter structures should be evaluated together through preoperative DTI and fMRI.

**Table 2:** Sulci of the parietal lobe and associated white matter tracts.

Sulci	White matter tracts^*^
Intraparietal sulcus	SLF II, MdLF, IFOF, Tapetum Optic Radiation^**^
Intermediate sulcus of Jensen	SLF II, MdLF, IFOF, Tapetum SLF III, dAF and Optic Radiation^†^
Post-central sulcus	SLF II, SLF III, AF

AF, arcuate fasciculus; dAF, dorsal arcuate fasciculus; IFOF, inferior fronto-occipital fasciculus; MdLF, middle longitudinal fasciculus; SLF, superior longitudinal fasciculus.

* Written superiorly to inferiorly.

** Optic radiation is only associated to IPS at the posterior most portion of it.

† SLF III, dorsal AF and optic radiation are not directly under the intermediate sulcus of Jensen but are in proximity.

**Table 3:** Anatomical structures and their functional importance and clinical manifestations in when damaged.

Anatomical structure	Functional importance	Clinical manifestation
Gyri		
Superior Parietal Lobe (BA 5 and 7) (Pilipović-Dragović et al., 2018; Türe et al., 1997)	Planned movement, spatial thinking, attention	Auditory or visual disconnection syndromes (hemifacial metamorphosia, tactile and visual anomia, auditory extinction, visual hallucinations)
Inferior Parietal Lobe (Supramarginal Gyrus [BA 40] &Angular Gyrus [BA 39]) (Salanova, 2018; Thiebaut de Schotten et al., 2011)	Perception of emotions in facial stimuli, interpretation of sensory information, language, mathematical operations, body image	Hemineglect, apraxia, acalculia, Gerstmann syndrome, visual field deficits
Postcentral Gyrus (BA 3b, 1 & 2) (Ribas, 2010; Türe et al., 1997)	Perception of sensation of contralateral body parts	Loss of sensation of contralateral body parts
Sulci		
Intraparietal Sulcus (Ribas et al., 2006)	Grasping and object recognition, understanding orientation in space, control of attention and eye movements, proprioception, arm-reaching movements, prehension	Speech and vision disorders
White matter		
SLF II (Ribas et al., 2006; Makris et al., 2005; Makris et al., 2009; Chechlacz et al., 2013; Mesulam, 1981; Thiebaut de Schotten et al., 2011; Türe et al., 2000; Türe et al., 1997; Schiff et al., 1983)	Identification of multiple items in space, execution of attentional tasks, visuospatial tasks, awareness, attention	Spatial hemineglect,Gertsmann Syndrome, visuospatial Neglect, spatial working memory disorders
SLF III (Ribas et al., 2006; Makris et al., 2009; Chechlacz et al., 2013; Türe et al., 1997; Heilman and Watson, 2008; Loui et al., 2009; Friederici et al., 2000; Noesselt et al., 2003; Schmahmann et al., 2007; Yagmurlu et al., 2016; Fridriksson et al., 2010; Glasser and Rilling, 2008; Duffau et al., 2003)	Articulation, phonology (dominant hemisphere), visuospatial tasks, awareness and attention, prosody, musical processing (non-dominant hemisphere)	Articulatory disorders (dysarthria/anarthria-dominant hemisphere), visuospatial Neglect (non-dominant hemisphere)
Ventral AF (Yagmurlu et al., 2016; Duffau et al., 2005; Duffau et al., 2008)	Phonologic processing of language (dominant hemisphere), perception and production of non-linguistic communication (non-dominant hemisphere)	Repetition disorder and phonemic paraphasia
Dorsal AF (Yagmurlu et al., 2016; Duffau et al., 2005)	Lexical and semantic language processing (dominant hemisphere), prosodic activation of language (non-dominant hemisphere)	Transcortical motor aphasia
IFOF (Glasser and Rilling, 2008; Duffau et al., 2003; Duffau et al., 2005; Duffau et al., 2008)	Lexical, semantic, and visuospatial processing, integration of the multimodal sensory input	Semantic parapaphasia
MdLF (Yagmurlu et al., 2015; Chan-Seng et al., 2014; Moritz-Gasser et al., 2013)	Language and attention, audiospatial processing	Failure to localize sound in Balint’s Sydrome (bilateral damage)
Optic Radiation (Duffau et al., 2008)	Vision	Quadranatopia (unilateral damage)

AF, arcuate fasciculus; IFOF, inferior fronto-occipital fasciculus; MdLF, middle longitudinal fasciculus; SLF, superior longitudinal fasciculus.

### Relation to the ventricular system

The meeting point where the POCS and the IPS meet is the closest point to the atrium and can be used as a surgical landmark for pathology located there (Ribas, 2010). In Figure 1D, one can see different types of IPS morphology. Morphology of IPS is crucial since its opening into POCS determines the location of the meeting point. If the IPS deviates laterally after arising at the end of the POS on the dorsolateral surface, this decreases the distance between the posterior sylvian point and the meeting point, while if the IPS deviates medially, the distance increases. When the distance between these two landmarks is short, there is increased probability of injury to the AF and OR, and a transcortical approach from the SPL or a transsulcal approach from the posterior IPS instead of an anterior transsulcal approach from the meeting point can be used. With this strategy, the possibility of injury to the AF and OR is reduced. On the contrary, the probability of the SLF II and SLF III passing under the meeting point increases when the distance between the posterior end point of the sylvian fissure and the meeting point lie closer to each other (when the meeting point is located more laterally). Thus, when surgeons are planning an approach to the atrium of the fourth ventricle, they should keep in mind that the relationship between the meeting point and the underlying AF, SLF II, and SLF III is quite variable; this variability could be predicted by taking the distance between a perpendicular line passing through the sylvian fissure and the meeting point into consideration. Although indirectly, it can be said that the morphology of the IPS determines the surgical approach.

In their study Dziedzic et al. (2021) studied cortical and subcortical anatomy of the parietal lobe and reported measurements of topographic landmarks of the parietal lobe. As they described in their study, approaches from anterior IPS are safe in 60% of the cases. As we demonstrated in our study, anterior end of the IPS is subject to variation and should not be chosen as the entry point in every case. If the anterior end of IPS locates laterally, the risk of injuring SLF II, arcuate fascicle, and optic radiation is higher. In the case of injury to these fibers, aphasia, dysphasia, visual defects can occur at the dominant hemisphere, visuospatial dysfunction, contralateral hemineglect syndrome can occur at the non-dominant hemisphere. Thus, posterior part of IPS is relatively stable topographical landmark and, in our opinion, it must be the choice of safe entry. We believe that the relationship of the lesion with subcortical white matter structures should be evaluated together through preoperative DTI and fMRI. Additionally, intraoperative neuromonitoring and/or awake craniotomy should be done to avoid damage to white matter tracts. Detailed description of topographical landmarks and corresponding white matter tracts are summarized in Table 2 and clinical manifestation in cases of injury to selected anatomical substrates are presented in Table 3.

## Conclusion

Variations in the complex anatomy of the lateral parietal lobe make this region a challenging area in which to safely operate. Recognition of the variations in surface and subcortical anatomy in the parietal region, and their relationship to eloquent brain tissue, will enable the surgeon to select safer surgical trajectories and minimize the incidence of complications.

## Data Availability

The original contributions presented in the study are included in the article/supplementary material, further inquiries can be directed to the corresponding author.

## Footnotes

^1^ https://db.humanconnectome.org

^2^ http://dsi-studio.labsolver.org

^3^ https://surfer.nmr.mgh.harvard.edu/

## Glossary

AF: Arcuate fasciculus
AnG: Angular gyrus
dAF: Dorsal arcuate fasciculus
DTI: Diffusion tensor ımaging
IFOF: Inferior fronto-occipital fasciculus
ILF: Inferior longitudinal fasciculus
IPL: Inferior parietal lobule
IPS: Intraparietal sulcus
ISJ: Intermediate sulcus of Jensen
ITG: Inferior temporal gyrus
MLF: Middle longitudinal fasciculus
MTG: Middle temporal gyrus
OR: Optic radiation
POCS: Postcentral sulcus
POS: Parieto-occipital sulcus
SLF: Superior longitudinal fasciculus
SmG: Supramarginal gyrus
SPL: Superior parietal lobule
STG: Superior temporal gyrus
STS: Superior temporal sulcus
vAF: Ventral arcuate fasciculus
